# Supplementary material for: Patterns of Intron Gain and Loss in Fungi
Source: PLoS Biol. 2004 Nov 30;2(12):e422. doi: 10.1371/journal.pbio.0020422 (PMC532390; doi:10.1371/journal.pbio.0020422)
Supplement: Table S1 — Also available at http://genes.mit.edu/NielsenEtAl/. (4.3 MB ZIP). [file pbio.0020422.st001.zip › NielsenEtAl/html/261.html]

AN9339.1.NCU00355.1.MG06442.1.FG06733.1


```
 CLUSTAL W (1.82) Multiple Sequence Alignments - Introns Inserted


Sequence 1: NCU00355.1	719 aa
Sequence 2: FG06733.1	716 aa
Sequence 3: AN9339.1	722 aa
Sequence 4: MG06442.1	727 aa
Alignment Length: 747 aa
Number Identitical Residues: 339 aa
Alignment Score (without introns) 17124


MG06442.1 	MPRSSMLGLWASLVWIAQAGCPYADPAALRPREEH------TDDYLAQFSVDDQDKFLTS
NCU00355.1	MRVNALLPLSG-LIGTALAACPFADPSALGRRAEGGEV--DARQRLKEVEVDDNGQFMTT
FG06733.1 	MVSSKLLVVLG-LASLTAAQCPYADPAKLAARDEG-----SSREHLKDYEVDDSKGHMSS
AN9339.1  	---MRALGLVG-LVGVANAVCPYMT-GELGRRDTNPDATEATEEFLSEYYLDDTDSYLTT
          	      * : . *   : * **:   . *  *    . :. : : * :  :**   .:::

MG06442.1 	DVGGPIGDQRSLKAGARGSTLLEDFVFRQKITHFDHER0VPERAVHARGAGAHGTFTSYG
NCU00355.1	DFGGNIEEQFSLKAGGRGSTLLEDFIFRQKLQHFDHER0IPERVVHARGAGAHGIFTSYG
FG06733.1 	DAGGPFEEQESLKAGERGPTLLEDFIFRQKITHFDHER0VPERAVHARGAGAHGYFKSYS
AN9339.1  	DVGGPIEDQQSLKAGARGSTLLEDFIFRQKIQRFDHER0VPERAVHARGAGAHGVFTSYG
          	* ** : :* ***** **.******:****: :***** :***.********** *.**.

MG06442.1 	DFSNITSASFLNSAGKRTPVFVRFSTVAGSRGSADSARDVHGFATRF2YTDEGNFD1IVG
NCU00355.1	DWSNITAASFLGAKDKQTPVFVRFSTVAGSRGSADTARDVHGFATRF2YTDEGNFD~IVG
FG06733.1 	DWSNITAASFLSSKDKNTPVFVRFSTVAGSRGSADTVRDVHGFATRF2YTDAGLFD~IVG
AN9339.1  	DFSNITAASFLSAEGKETPVFVRFSTVAGSRGSSDLARDVHGFATRF~YTDEGNFD~IVG
          	*:****:****.: .*.****************:* .********** *** * ** ***

MG06442.1 	NNVPVFFIQDAIQFPDLVHAVKPSPDKEIPTAATGKRPDYKPNPDAT1HDSAWDFFSQQS
NCU00355.1	NNIPVFFIQDAIRFPDLIHSVKPSPDNEVPQAAT------------A~HDSAWDFFSSQP
FG06733.1 	NNIPVFFIQDAIQFPDLIHAVKPSPDSEIPQAAT------------A~HDSAWDFFSQQT
AN9339.1  	NNIPVFFIQDAIQFPDLIHAVKPKGDREIPQAAT------------A~HDAAWDFFSQQP
          	**:*********:****:*:***. * *:* ***            : **:******.*.

MG06442.1 	2SSLHALFWAMSGYGIPRSYRHMDGFGVHTFRMVADNGESRLVKFHFKTKQGKASLIWEE
NCU00355.1	~SALHTLFWAMSGNGIPRSYRHMDGFGIHTFRLVTEDGKSKLVKWHWKTKQGKAALVWEE
FG06733.1 	~TTLHTLFWAMAGYGIPRSFRHVDGFGVHTFRLVTDEGDTKFVKWHWKSKQGKASLVWDE
AN9339.1  	~STLHTLLWAMAGHGIPRSFRHVDGFGVHTFRLVTEDGSTKLVKFHWKTLQGLASMVWEE
          	 ::**:*:***:* *****:**:****:****:*:::*.:::**:*:*: ** *:::*:*

MG06442.1 	AQVLSGKNPDFHRQDLWDAIEAGHGPEWE~FGVQVVNEEDALRFGFDMLDPTKILPEDLV
NCU00355.1	AQVLAGKNADFHRQDLWDAIESGNAPSWE~LAVQLIDEDKAQAYGFDLLDPTKFLPEEFA
FG06733.1 	AQHLAGKNADYHRADLWNAIESGNGPEWE~LNAQIFDEDQALAFGFDVLDATKIIPEELA
AN9339.1  	AQQISGKNPDYMRQDLFESIEAGRYPEWE0LNVQIMDEEDQLRFGFDLFDPTKIVPEEYV
          	** ::***.*: * **:::**:*. *.** : .*:.:*:.   :***::*.**::**: .

MG06442.1 	PVHPLGLLKLDRNPINYFAETEQIM0FQPGHLVRGIDFTEDPLLQ~-GRVFSYLDTQLNR
NCU00355.1	PLQVLGEMTLNRNPMNYFAETEQIS~FQPGHIVRGVDFTEDPLLQ~-GRLYSYLDTQLNR
FG06733.1 	PLQPIGVMKLDTNPVNYFAETEQVM0FQPGHIVRGIDFTEDPLLQ~-GRIFSYLDTQLNR
AN9339.1  	PLTPLGKMTLNRNPRNYFAETEQVM0FQPGHVVRGVDFTEDPLLQ0QGRLFSYLDTQLNR
          	*:  :* :.*: ** ********:  *****:***:********* .**::*********

MG06442.1 	HNGPNFEQ1LPINRPRVTVHNNNRDGSGQNFIHKNIHPY1T----PSALN-EFPKQADDD
NCU00355.1	HRGPNFEQ~LPINRPVSGVHNNHRDGQGQAWIHKNIHHY~S----PSYLNKGYPAQANQT
FG06733.1 	HGGPNFEQ~LPINRPLSPIHNNNRDGAGQNFIHKNTAAS1DSLDTPNTLNKGYPVQANQT
AN9339.1  	NGGPNFEQ~LPINQPRVAIHNNNRDGAGQMFIPLNPDAY~S----PNTLKGSTLKQANQT
          	: ****** ****:*   :***:*** ** :*  *          *. *:     **:: 

MG06442.1 	QGRGFMTAPRRRTQDAALVRELSETFDDHWTQPRLFWNSITPAEQQILVNAIRFETSNIV
NCU00355.1	VGRGFFTTPGR-TASGVLNRELSATFDDHYTQPRLFFNSLTPVEQQFVINAIRFEASHVT
FG06733.1 	QGKGFFTAPGR-KVSGNLVRARASTFSDHWSQPRLFYNSLTKVEQQFLINAIRFETSQLM
AN9339.1  	AGRGFFTAPDR-TANGNLVRAKSSTFDDAWSQPRLFWNSLLPAEKQFVVNAIRFENANVK
          	 *:**:*:* * . .. * *  : **.* ::*****:**:  .*:*:::****** ::: 

MG06442.1 	NTHVRQKVLQQLNRISDELAGRVAQALGMEAPAPEPAFYHENTTIGLSIFGIKLPTIKTL
NCU00355.1	NEQVKKNVLEQLNKISNDVAKRVAVALGLEAPQPDPTYYHNNVTRGVSIFNESLPTIATL
FG06733.1 	SETVQKNVLYQLNKISHDIAVRVGRALGLKAPEADDKYYHDNTTAGISIFGTKLPSIATL
AN9339.1  	SDVVKNNVIVQLNRISNDLATRVAKAIGVDAPEPDNTYYHDNTTSNIGAFGHRLQSLAGL
          	.  *:::*: ***:**.::* **. *:*:.** .:  :**:*.* .:. *.  * ::  *

MG06442.1 	RVGVLASTGNVASLSQAADLKARFMGDGVAVAVLGETQVEGVDVTYSQSDATVFDGIIIA
NCU00355.1	RVGVLSTTKGG-SLDKAKALKEQLEKDGLKVTVIAEYLASGVDQTYSAADATAFDAVVVA
FG06733.1 	SVGVLVSINSNNSIQQAKALKKALGEDKVTVSIIGEVLGDDVEVTYSAATAAQFDSVVVT
AN9339.1  	KIAVLASVDAEESFSAATALKAELSNDNLDVIVVAERFSNGVNQTYSASDAIQFDAVVVA
          	 :.** :     *:. *  **  :  * : * ::.*   ..*: *** : *  **.::::

MG06442.1 	AGAEGLVAKDAP---ASALFPIGRPQQLVVDSFRWGKPIGLAAASDDSANIFSSLGTTGP
NCU00355.1	EGAERVFSGKGA---MSPLFPAGRPSQILTDGYRWGKPVAAVGSAK-KALQSIGVE-EKE
FG06733.1 	SGTESLFNGTG----KSTLFPPGRPTQIIVDGYHWGKPVGFIGGAK-AAAQAARVS-KGK
AN9339.1  	PGAEKLFGAKSAANSSSTLYPAGRPLEILVDAFRFGKPVAALGSGS-TAFDNAGINTAVE
          	 *:* :.   ..:.: *.*:* *** :::.*.:::***:.  ....  *     : :   

MG06442.1 	GVFSGDDLGVLVTD-FKEGLSTYKFVERFPMDS----
NCU00355.1	AGVYAGAQDEVIKG-VEEGLKVFKFLERFAVDGDDEE
FG06733.1 	GVYFPKNVNSLVED-LKDGLATFKFTDRFPLDH----
AN9339.1  	GVYVADAVDESFANNLEEGLTVFKFLDRFALDSDE--
          	.       .  . ...::** .:** :**.:* ..
```
